# Disentangling the Long-term Effects of Divorce Circumstances on Father–Child Closeness in Adulthood: A Mediation Analysis

**DOI:** 10.1007/s10680-022-09636-1

**Published:** 2022-10-19

**Authors:** Juul Spaan, Ruben van Gaalen, Matthijs Kalmijn

**Affiliations:** 1 Netherlands Interdisciplinary Demographic Institute (NIDI)-KNAW/University of Groningen, Lange Houtstraat 19, 2511 CV The Hague, The Netherlands; 2 Statistics Netherlands, PO Box 24500, 2490 HA The Hague, The Netherlands; 3 University of Amsterdam, PO Box 15508, 1001 NA Amsterdam, The Netherlands

**Keywords:** Family complexity, Nonresidential fathers, Intergenerational relations, Interparental conflict, Fathers’ involvement

## Abstract

Many studies have shown that the relationship between nonresidential fathers and their children in youth has a lasting influence on their relationship in adulthood. Comparatively less is known about the process through which divorce affects father–child relationships. We assess if and how the divorce circumstances of interparental conflict, the presence of new partners, and geographical distance between parents affect nonresidential father–child closeness in adulthood. Using a path model, we test whether father–adult child closeness is mediated by fathers’ involvement after divorce. The results of this study demonstrate that the level of interparental conflict and the presence of a fathers’ new partner after the divorce negatively affect the closeness between fathers and children in adulthood. Our mediation analysis demonstrates that both the effects of interparental conflict and new partnerships on closeness are partially mediated by father involvement and contact frequency during childhood. In other words, it is partly through the negative effect that interparental conflict and new partners have on fathers’ involvement that fathers and children become less close later in life. Our study highlights the importance of disentangling the effects of different factors associated with divorce when examining nonresidential father–child relationships.

## Introduction

Past research has demonstrated the importance of fathers’ involvement for children’s outcomes (Dunn, 2004). Father–child time improves children’s cognitive abilities and is associated with better academic outcomes for children of all socioeconomic backgrounds (Cano et al., 2019; Miller et al., 2020; Modecki et al., 2015). As a result of increased divorce rates since the second half of the twentieth century, an increasing number of children grow up with a nonresidential father. Previous studies have documented a negative association between nonresidential parenthood and father involvement (Fransson et al., 2018; Grätz, 2017; Köppen et al., 2018; Ryan et al., 2008; Seltzer, 1991; Stephens, 1996). Moreover, it has been found that divorced fathers’ involvement declines over time (Haux & Platt, 2021; Stephens, 1996), even though in recent decades fathers have expressed a growing desire to be involved in their children’s lives (Kaufman, 2013). This is of concern, because it may have long-term implications for the father–child relationship.

Many studies have demonstrated that nonresidential fathers’ involvement in youth has a lasting influence on their relationship in adulthood. For example, low levels of divorced fathers’ involvement are associated with reduced contact frequencies and lower reports of relationship quality in father–child bonds in adulthood (Aquilino, 2006; Kalmijn, 2015b; King, 2002; King & Sobolewski, 2006). Comparatively less is known about the process through which divorce affects father–child relationships. There is some evidence that the circumstances and decisions made in the period following divorce, which we will refer to as divorce circumstances, have an impact on father involvement. One of these factors is the level of conflict between the parents in youth. Studies have demonstrated that conflict between parents is negatively associated with parental cooperation (Toews & McKenry, 2001), which could affect nonresidential parents’ access to their children. New partnerships may influence father involvement as well because it requires fathers to renegotiate the tie to the ‘original’ family (Aquilino, 2006; Manning & Smock, 2000; Seltzer, 1991). Upon divorce, there is a transition from all members residing in the same location to children living at a distance to one of the parents. A greater geographical distance requires more coordination and shapes the possibilities for father–child interaction (Stjernström & Strömgren, 2012). Considering that investments in children’s youth are reciprocated later in life (Silverstein et al., 2002a, 2002b), and given the continuity of the father–child relationship (Aquilino, 2006; Kalmijn, 2015a), a question that arises is whether circumstances surrounding the divorce affect closeness in adulthood in part because they either limit or enhance fathers’ possibilities to be involved after divorce.

The first aim of this study is to get a better understanding of the long-term effects of divorce circumstances on how close fathers and children are in adulthood. We do so by first assessing if and how three important divorce circumstances—interparental conflict, the presence of a new partner, and the geographical distance between parents—affect fathers’ involvement and father–child contact frequencies immediately after divorce. Then, we examine the effect of divorce circumstances on nonresidential father–child closeness in adulthood. Finally, we examine how we can explain these effects using standard decomposition methods, and test whether the effects of the divorce circumstances on adult closeness are mediated by fathers’ involvement and contact with the children after divorce. If we find evidence for mediation along these lines, this would suggest that the long-term effects of divorce on the father–child relationship are shaped early in the life course. For example, by remaining geographically close to a child after divorce, the father–child post-divorce relationship will remain strong and this leads to a close relationship when the child is adult and independent. In contrast, by moving further away after the break-up, the father may lose contact in the years after divorce and the long-term relationship with the child is compromised.

The second aim of this study is to compare the effects of the quantity and quality of father–child interaction. An ongoing debate in the literature is whether it is the quantity or the quality of the time spent together that matters for the parent–child relationship. Two meta-analyses that compared the effects of the quantity of face-to-face contact and the quality of father involvement on children’s wellbeing found that there is no association with contact frequency, but a positive association with involvement, and concluded that contact in and of itself is not sufficient for a beneficial effect on child wellbeing (Adamsons & Johnson, 2013; Amato & Gilbreth, 1999). Most studies examining the relationship between parents and children following divorce focus on either contact frequency or involvement related measures. Analyzing both aspects of father involvement simultaneously increases our understanding of the difference between these two operationalizations of fathers’ roles in children’s upbringing after a divorce.

This research is of importance because of the increased probability that children grow up with a nonresidential parent. This study combined rich retrospective survey data from the Parents and Children in the Netherlands survey (Kalmijn et al., 2018) with detailed register data on parents’ geographical location from the teenage years of the respondents, retrieved from the System of Social-statistical Datasets of Statistics Netherlands (Bakker et al., 2014). In the Netherlands, nowadays three in ten children aged fifteen are not registered with both parents at the same address (Statistics Netherlands, 2018). After a divorce, the majority of children resides with the mother, and even for those registered with the father, a part has a different residential arrangement, such as shared residence (Poortman & van Gaalen, 2017; van der Wiel & Kooiman, 2019). Furthermore, the Netherlands, like many other European countries, faces challenges with an aging population. Together with lower expenses on welfare, family ties, such as the parent–child relationship, are becoming even more important as a source of support in old age. This highlights the importance of understanding how changes in children’s circumstances as a result of increased family complexity while growing up affect the parent–adult child relationship in the long run.

## Theoretical Background

### The Opportunity Structure of Interaction

This study is set in the context of the life course perspective. Studies of the life course emphasize the interconnectedness of individual family members’ life paths and the continuity of the relationship over time (Elder et al., 2003). Based on the principle of linked lives, the assumption is that early transitions in the lives of parents and children play an important role in the parent–child relationship in adulthood (Elder, 1994). Traditionally, the parent–child relationship is considered an exchange relationship of care and support between family members of different generations, otherwise known as intergenerational solidarity (see Bengtson & Roberts, 1991). In this framework, the focus shifts from the nuclear family to multigenerational bonds (Bengtson, 2001). According to Bengtson and Roberts (1991), how well people can participate in exchanges between family members is influenced by the opportunity structure of family interaction, which consists of structural components, such as proximity to family members, their health, and the number of family members. A divorced family is particular in that it has some characteristics of both nuclear family structures and adult families: On the one hand, similar to an intact family, it is mainly a one-way relationship; parents’ investments are not (yet) reciprocated. On the other hand, when children of divorce reside with only one of the parents, their tie to the nonresidential parent is similar to parents and children in adult families, because they no longer share a household. Therefore, nonresidential parents and children face similar constraints as adult family members in terms of the opportunity structure of interaction. Applying the intergenerational solidarity perspective to divorced families could provide further insights into nonresidential parent–child relationships.

From an intergenerational solidarity perspective, parental involvement in youth has been demonstrated to partly function as an implicit social contract that ensures the lifelong exchange of care and support. Using longitudinal data from the U.S., Silverstein et al. (2002a, 2002b) showed that parents who spent more time doing activities with their children in youth received more support later from their grown-up children. Put differently, the level of intergenerational solidarity between parents and children in adulthood partly results from investments made in the parent–child relationship earlier in the life course. A divorce limits parents’ ability to invest in their children, as the time that a child could spend with both parents simultaneously before the divorce after the divorce is spent with only one parent. In addition, financial transfers are possibly limited as a result of the negative financial consequences associated with divorce (Andreß et al., 2006). Particularly nonresidential parents’ reduced opportunities to invest in their children, as a result of structural changes, carry on over the life course and may alter the parent–child relationship. Prior studies have demonstrated that nonresidential fathers who were less involved in childhood had a lower relationship quality with their adult children (Aquilino, 2006; Kalmijn, 2015a; King, 2002; King & Sobolewski, 2006). We, therefore, expect that children whose fathers were less involved (hypothesis 1a) or who saw their fathers less frequently in youth (hypothesis 1b) are less close to their fathers in adulthood.

### Interparental Conflict

Whether nonresidential fathers can stay involved in their children’s lives may depend on their ability to cooperate with their ex-partner. Parents’ ability to cooperate has been demonstrated to be negatively associated with the level of conflict. Divorced parents who had high levels of conflict were found to be less cooperative, for example in terms of making decisions and discussing parenting-related matters (Toews & McKenry, 2001). In contrast, cohesive parenting is associated with increased contact frequency with children (Viry, 2014), and mothers who perceived the parenting alliance to be strong, reported more involved nonresidential fathers (Futris & Schoppe-Sullivan, 2007). Furthermore, high levels of conflict between parents have been associated with feelings of being caught between parents (Amato & Afifi, 2006; Buchanan et al., 1991). For children whose parents fought frequently, there might be more pressure to take sides and to distance themselves from the nonresidential parent. Indeed, children who were pressured to side with their mother, also known as “gatekeeping”, have been shown to have less frequent contact with their nonresidential father (Walper et al., 2020). Previous studies found that high levels of interparental conflict function as a barrier to nonresidential fathers’ involvement (Ryan et al., 2008). Therefore, we expect that high levels of interparental conflict have a negative effect on fathers’ involvement in youth after the divorce (hypothesis 2a), and lead to lower contact frequencies (hypothesis 2b).

The level of conflict between parents may have a lasting influence on the parent–child relationship. For children with married parents, spill-over effects of the interparental conflict have been reported, with higher levels of interparental conflict resulting in a less cohesive and more conflictual or negative environment for the family as a whole (Gerard et al., 2006; Katz & Woodin, 2002; Schoppe-Sullivan et al., 2007). In divorced families, too, a negative association between interparental conflict and parent–child relationship quality has been reported, both in youth as well as in adulthood (Amato & Booth, 1996; Dunn et al., 2005; Uphold-Carrier & Utz, 2012). However, there has been some evidence that a divorce could have a stress-relieving effect (Wheaton, 1990). The parental divorce could be an opportunity for children to see their parents separately, enabling children to build a personal relationship with less influence from the quality of the relationship between parents. It has been reported that by no longer being in a dysfunctional marriage, a more calm environment may emerge, which could have positive effects on the parent–child relationship (Kalmijn, 2015a; Yu et al., 2010). However, considering that these findings concern a moderating effect, we do not expect that the stress-relieve effect outweighs the negative consequences of a high-level conflict divorce. Rather, we expect high levels of interparental conflict in youth to lead to less close relationships between nonresidential fathers and children in adulthood (hypothesis 2c), but that this effect is mediated by post-divorce father involvement and contact frequency (2d).

### New Partnerships

Nonresidential fathers may form new partnerships after divorce, as they reenter the partnership market as single parents (Thomson, 2017). Especially when fathers start living together with the new partner, children gain a stepparent. However, when children do not share a residence with this father, this stepparent remains at a distance. Fathers who have a new partner have to distribute their time among more people, which could come at the cost of fathers’ involvement and contact frequencies with their children. There is mixed evidence regarding the effects of new partnerships on fathers’ involvement; some studies demonstrated that remarriage leads to reduced contact frequencies with children (Bradshaw et al., 2002; Seltzer, 1991; Stephens, 1996; Stewart, 1999, 2010), while others found no significant effect (Aquilino, 2006), or only an effect when the new union followed closely upon separation (Juby et al., 2007). The shift in focus from nonresidential children to new residential children has been characterized as “swapping families” (Manning & Smock, 2000; Tach et al., 2014; Townsend, 2002). Previous studies found that when fathers had children with a new partner or lived with their stepchildren, frequencies of fathers’ visits to nonresidential children were lower and there was less financial support (Juby et al., 2007; Manning & Smock, 2000; Manning et al., 2004). We, therefore, expect new partnerships to reduce fathers’ involvement (hypothesis 3a), and to result in lower contact frequencies (hypothesis 3b).

Similar processes could affect how close fathers and children are in adulthood. Fathers who remarried have been demonstrated to receive less support from their adult children (Kalmijn, 2007), and were reported to have less frequent contact, fewer exchanges of support, and a lower relationship quality (Kalmijn et al., 2019; Kalmijn, 2015b; Noël-Miller, 2013). In line with the “swapping families” perspective in youth (Manning & Smock, 2000), it has been found that when fathers repartner, their new partner facilitates the relationship to stepchildren, but not to the father’s biological children in adulthood (Kalmijn et al., 2019). Therefore, we expect new partnerships to lead to the reduced closeness between fathers and adult children (hypothesis 3c), but that this effect is mediated by postdivorce father involvement and contact frequency (3d).

### Geographical Distance

A structural circumstance that changes as a result of divorce is the geographical proximity between parents and children. The transition from being married to being divorced requires at least one of the ex-partners to move out of the shared residence, which results in a physical distance between the ex-partners. Although nonresidential parents can continue to play an important role in their children’s lives despite not living in the same house, the geographical distance between two parents shapes the possibilities for contact between the nonresidential parent and the child. Logistically, when there is a greater geographical distance, the longer commute may take up time that could otherwise be spent together, it requires better planning and reduces the opportunities for spontaneous meetings. Nonresident fathers have been reported to spend less time on children’s day-to-day live activities, and more time on recreational activities (Amato & Gilbreth, 1999; Stewart, 1999). Furthermore, fathers who live close by may benefit from their children’s active efforts in maintaining contact. Depending on the age of the child, certain distances can be bridged by the child itself, but larger distances might make this much less feasible. Previous studies found that great geographical distances are negatively associated with the frequency of visits and contact (Arditti & Keith, 1993; Cooksey & Craig, 1998; Furstenberg et al., 1983; Seltzer, 1998; Viry, 2014). Therefore, we expect that nonresidential fathers who live at a greater distance from their children are less involved in their children’s lives (hypothesis 4a), and have lower contact frequencies (hypothesis 4b).

In adulthood, the distance to nonresidential fathers in youth could play a similar role with regards to the father–child relationship. To our knowledge, no research has examined the relationship of proximity in youth to closeness in adulthood. Previous studies have examined duration of co-residence and found that fathers who lived together with their children for a shorter period of time prior to divorce, were less close to their adult children (Kalmijn et al., 2019). Reduced opportunities to invest in the child are offered as a possible explanation. In case of a greater geographical distance, there might be a similar mechanism at work, with parents who live further away facing more difficulties to invest in the parent–child relationship. The reduced frequency of visits and contacts in youth cited earlier supports this line of thinking. Therefore, we expect that nonresidential fathers who lived further away from their children after the divorce are less close to their children in adulthood (hypothesis 4c), but that this effect is mediated by postdivorce father involvement and contact frequency (4d).

### Control Variables

We included some measures that could be associated with father involvement and contact frequency in youth. One factor is gender, as sons and daughters might evaluate the relationship with their father differently (Fingerman et al., 2020; Kalmijn, 2007, 2015b; King, 2002, 2006; Willson et al., 2003). Past research indicates that the sibling structure (Fingerman et al., 2009; Kalmijn, 2013; Silva et al., 2009; Spitze & Logan, 1991) and the age at which a child experiences the divorce (Aquilino, 2006; Kalmijn, 2015a) affect intergenerational ties as well. We further consider the fathers’ level of education, because more resourceful parents may be more aware of the negative effects of divorce and may be more effective in working out arrangements to avoid damaging effects on children (Bradshaw et al., 2002; Stephens, 1996). The current relationship between fathers and adult children might be affected by some additional factors, such as the age of the respondent. Older respondents have to look back further in time when answering questions about their childhood, which could result in biased reports as experiences that occurred later in life might influence the evaluation of the past (de Vries & de Graaf, 2008; Karney & Coombs, 2000). Finally, we include some characteristics of the greater family network, which, in line with the linked live perspective, have been shown to affect the parent–child relationship, such as the anchor’s life course stages of being partnered and having children (Kalmijn, 2007, 2015b; Min et al., 2022; Ward et al., 2014), and whether the mother had a new cohabiting relationship (King, 2006).

## Methods

### Data and Sample Selection

For this analysis, we employed data from the first wave of the Parents and Children in the Netherlands survey (OKiN), a survey conducted in 2017 among a stratified random sample of Dutch adult children aged 25 to 45 and their parents (Kalmijn et al., 2018). People who grew up in non-standard families were systematically oversampled based on whether the adult child was registered with both parents at age 15 or not. This resulted in a sample of 6845 persons (response rate of 62%), of which 33% grew up in a nonintact family without a new partner, and 42% in a family with a new partner. The large proportion of people growing up in new partner families ensured that there were a sufficient number of stepparents in the data. Via the System of Social-statistical Datasets of Statistics Netherlands (Bakker et al., 2014), residence coordinates based on address data from the anchors’ parents from the years 1995 to 2018 were linked to the data. Due to the address data being available from 1995 onwards rather than from 1971, when the first respondents were born, not all birth cohorts from the survey could be included, but the analytical sample did cover all child ages at which a divorce occurred.

For this analysis, we focused on the 3390 so-called anchors who grew up with divorced or separated parents who were in a heterosexual relationship. As it concerns the dissolution of a partnership through both divorce and separation, we use one term to cover both. We excluded respondents with a deceased parent (*n* = 688), and, because of our focus on nonresidential father–child relationships, those who had a different parenting arrangement from a resident mother arrangement. This concerns children who lived only with the father after the divorce (*n* = 328), children whose parents had a coparenting arrangement (*n* = 160), or children with another arrangement (*n* = 65). In these cases, different mechanisms might be at work than for resident mother arrangement, which require in-depth analyses in itself. To ensure that the analyses were comparable across the models, we harmonized the data to include anchors who had no missing values on closeness in adulthood (*n* = 201). The analytical sample consisted of *n* = 1948 respondents.

### Measures

The first main independent variable of interparental conflict was measured by the anchor’s report of the frequency of conflict between parents in the period before divorce on a scale from (1) never to (4) often. A scale was calculated from respondents’ responses to three statements: (1) there were tensions and/or conflicts between your parents, (2) your parents did not want to talk to each other for a while, and (3) there were serious fights between your parents. We focused on the period before divorce rather than the post-divorce level of conflict, because marital conflict is less endogenous. For example, high levels of post-divorce conflict could be the result of fathers not being allowed to see their children, but not necessarily reflective of the parents’ relationship quality. Even though the divorce might relieve some of the tensions between parents, past research has shown that conflicts during marriage affect the post-divorce relationship between former spouses (Fischer et al., 2005).

For the second main independent variable, new partnerships, respondents were asked about the presence of a new partner with whom the father had a cohabiting relationship during the anchor’s youth, which we dummy-coded to (0) had no new partner and (1) had a new partner.

The third main independent variable was the geographical distance between separated parents. The air-line distance in kilometers was calculated based on the residence coordinates of the parents’ residences on the 1st of October of each year. We calculated the mean of known distances between the ages of 15 to 18. An advantage of focusing on a 3-year period is that the distance is not based on a single point in time, but is reflective of a longer period. In our models, we included the dummy variable for parents living within 10 km distance of each other (0) and parents living more than 10 km apart (1). The 10 km cutoff point has the most substantive meaning in the Dutch context: this distance is quite short for parents with a car but is also still accessible for children to reach independently, for example by bike or public transport.

The first mediating variable was fathers’ involvement with children after the parental separation. This scale variable was based on five items asking the respondents retrospectively how frequently their father carried out various activities after the parental divorce on a scale from (1) very often, (2) often, (3) sometimes, (4) (almost) never, and (5) never (activity not done). The latter two categories were combined into one category of (4) (almost) never, because both indicate that this time was not spent together. The activities were (1) talked with you about school or education, (2) helped you with homework and school assignments, (3) talked with you about personal issues, (4) took you out on days out or participated in hobbies with you, and (5) took you to or participated together with you in sports activities. We calculated the mean of the items with valid responses, with a value of 4 being coded to indicate the highest level of involvement. This scale variable had a Cronbach’s coefficient of *α* = 0.81.

The second mediator was an ordinal variable of face-to-face contact frequency in youth between fathers and children after the divorce. Children were asked “How often did you see your father after the separation?” with answer categories (1) never, (2) less than monthly, (3) about monthly, (4) about weekly, and (5) about daily. The respondent answered this question for each change in living situation. We included the response to contact frequency in the latest living situation up until age 14. Although our distance variable was measured at age 15 to 18, we did not include the living situation at these ages, because children from divorced families have a higher risk of very early home leaving, at age 15 and 16 (Goldscheider & Goldscheider, 1998). To enhance the consistency and coherence of the paper, we have chosen to transform our contact frequency variable into a log-linear variable, matching other studies focusing on contact frequency (Waite & Harrison, 1992). We assigned values to the endpoint of each category and recoded the 5 scale variable into and recoded the 5-point variable into (0) never, (12) monthly, (52) weekly, and (365) daily. We added 1 to each value and took the logarithmic value.

The main dependent variable was current closeness as reported by the anchor. Respondents were asked how close they were with their father at the time of the survey on a five-point scale of (1) very close to (5) not close at all. This was recoded such that a positive effect reflected a higher level of closeness. As in previous research, we treated this variable as a continuous variable in the analyses (Kalmijn et al., 2019; King, 2006).

We include a number of control variables that have been shown to be associated with intergenerational ties in a temporal fashion, meaning that in the youth models we include only control variables that occurred prior to the divorce, and the other relevant control variables in the adulthood models. In the youth models, we included gender, the number of full biological siblings of the anchor, the anchor’s age at separation, and the father’s highest attained level of education. We grouped age at separation into two age groups: age 0–6, and age 7–17, because previous studies found a significant difference between these two age groups (Aquilino, 2006; Kalmijn, 2015a). We recoded the father’s level of education into three groups according to the International Standard Classification of Education 2011 (Statistics Netherlands, 2011). The first group contains primary and lower secondary education, the second group upper secondary, post-secondary non-tertiary, and short-cycle tertiary education, and the third group bachelor, master, or doctoral or equivalent education. In the adulthood models, in addition to the abovementioned control variables, we included the current age of the anchor centered around the mean; whether the anchor had a partner and/or children, both dichotomized; and the anchor’s highest attained level of education, coded identical to the father’s level of education. We finally controlled for whether the mother had a new cohabiting relationship during the anchor’s youth or not. The descriptive statistics for all variables in the analysis can be found in Table 1.

**Table 1:** Descriptive statistics of variables. *Source*: OKiN Anchor 1.1 UvA 2017, own calculations

	Count	Missing	Mean	SD	Min	Max
*Dependent variables*						
Post-divorce father’s involvement in youth	1948	0%	1.7	0.6	1	4
Post-divorce contact frequency in youth (logged)	1948	0%	3.2	1.3	0	5.9
Post-divorce contact frequency in youth						
Never (0)	128		6.6%			
Less than monthly (3)	214		11.0%			
About monthly (12)	488		25.1%			
About weekly (52)	1042		53.5%			
About daily (365)	76		3.9%			
Closeness with father in adulthood	1948	0%	2.9	1.3	1	5
Not close at all	387		19.9%			
Not very close	350		18.0%			
Reasonably close	487		25.0%			
Close	501		25.7%			
Very close	223		11.4%			
*Key independent variables*						
Level of interparental conflict before divorce	1613	17.2%	2.3	1.0	1	4
Father’s new partnership status in youth	1861	4.5%				
No new partner	504		27.1%			
New partner	1357		72.9%			
Distance between parents in youth	1832	6.0%	21.9	32.5	0	244.7
10 km or less	1010		55.1%			
Over 10 km	822		44.9%			
*Control variables*						
Anchor’s gender	1948	0%				
Male	877		45.0%			
Female	1071		55.0%			
Anchor’s number of siblings	1948	0%	1.3	1.1	0	11
Anchor’s age at separation	1948	0%	7.5	4.0	0	17
Age 0–6	860		44.2%			
Age 7 or older	1088		55.9%			
Anchor’s age	1948	0%	32.1	5.2	25	46
Anchor’s current partnership status	1526	21.7%				
No partner	170		11.1%			
Has a partner	1356		88.9%			
Anchor’s own children	1948	0%				
No children	951		48.8%			
Has children	997		51.2%			
Anchor’s level of education	1948	0%				
ISCED 0–2	382		19.6%			
ISCED 3–5	871		44.7%			
ISCED 6–8	695		35.8%			
Father’s level of education	1467	24.7%				
ISCED 0–2	674		45.9%			
ISCED 3–5	356		24.3%			
ISCED 6–8	437		29.8%			
Mother’s new partnership status in youth	1844	5.3%				
No partner	552		29.9%			
Has a partner	1292		70.1%			
Observations	1948					

Note. Some deviations may occur due to rounding

### Analysis Plan

**Fig. 1:**
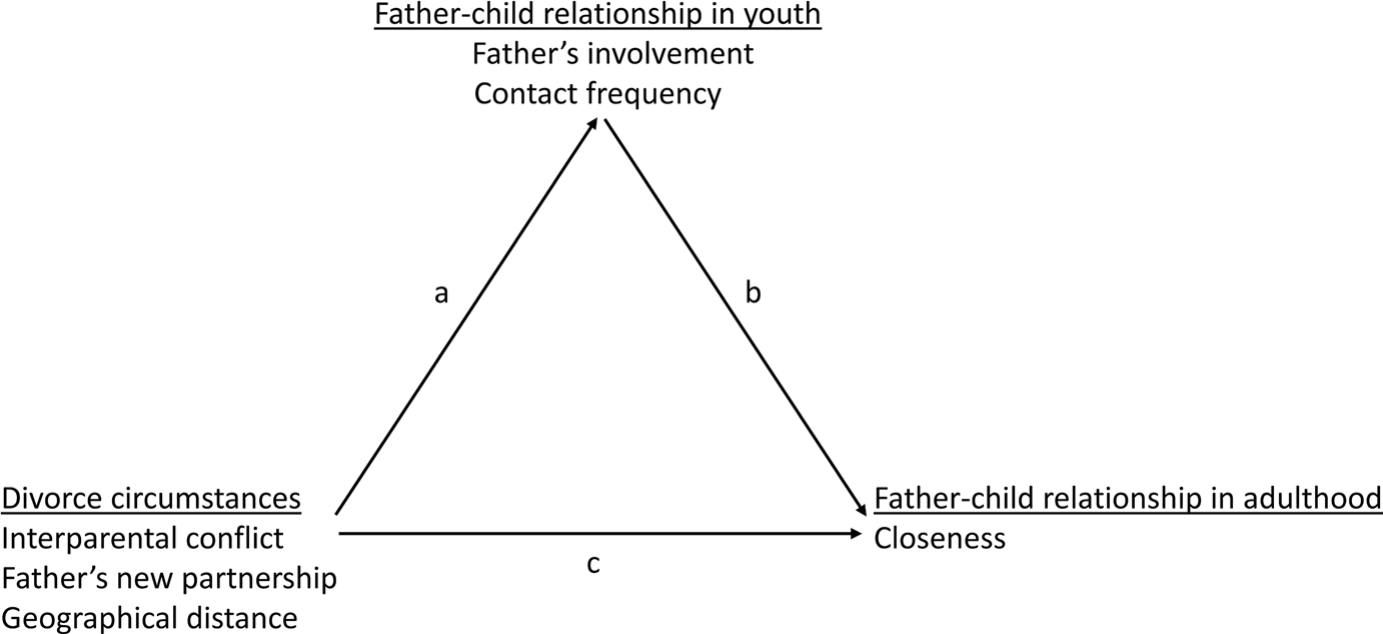
Diagram of mediation analysis

Figure 1 illustrates our mediation model. Following Baron and Kenny (1986), we considered two causal paths for the outcome variable of closeness between fathers and children in adulthood: the direct impact of the independent variables (path c), and the impact of the mediator (path b). In addition, there is a path that runs from the independent variables to the mediators (path a). A variable is considered a mediator when there is a significant association in path a and b, and when a previously significant association in path c is significantly reduced when paths a and b are controlled for

In Table 2, we examined the effect of divorce circumstances on the two mediator variables of father involvement and father–child contact after divorce to assess whether variations in the levels of the independent variables significantly account for variations in the mediator variables (path a). We estimated OLS models to examine the direct effect of the divorce circumstances on fathers’ involvement (Model 1) and father–child contact frequencies in youth (Model 2).

**Table 2:** Estimates from OLS models of divorce circumstances on nonresidential fathers’ involvement and contact frequency in youth. *Source*: OKiN Anchor 1.1 UvA 2017

	Model 1	Model 2
Interparental conflict before divorce	−0.157**	−0.275**
	(−10.23)	(−8.43)
Father's new partner in youth (ref = none)		
Had a new partner	−0.096**	−0.149*
	(−2.97)	(−2.25)
Distance between parents (ref. = less than 10 km)		
Over 10 km	−0.050^~^	−0.328**
	(−1.68)	(−5.20)
Gender (ref. = female)		
Male	0.038	0.043
	(1.35)	(0.76)
Anchor’s number of siblings	−0.030*	0.014
	(−2.26)	(0.52)
Age at separation (ref. = age 0–6)		
Age 7 or older	−0.058*	0.091
	(−1.97)	(1.51)
Father's level of education (ref. = ISCED 0–2)		
ISCED 3–5	0.108**	−0.129
	(2.82)	(−1.47)
ISCED 6–8	0.206** (5.09)	−0.104
		(−1.26)
Mother's new partner in youth (ref. = none)		
Had a new partner	−0.014	−0.030
	(−0.42)	(−0.45)
Constant	2.174**	4.075**
	(34.17)	(30.59)
N	1948	1948

*t* statistics in parentheses

Note. Father’s involvement was measured as a continuous variable from never (1) to often (4) and contact frequency in youth was measured as the logarithm of the transformed categorical variable from 0: never, 4: less than monthly, 12: about monthly, 52: about weekly, 365: about daily

^~^*p* < 0.10, *p < 0.05, **p < 0.01

In Table 3, we estimated OLS models to assess the effects of fathers’ involvement (Model 1) and contact frequency (Model 2) on closeness in adulthood, i.e., the effects of the mediators on closeness (path b). In this table, we also examined the effects of the divorce circumstances (Model 3) on closeness in adulthood without controlling for the mediators (path c). These effects represent the total effects of the divorce circumstances.

In Table 4, we used the KHB method to estimate mediation effects (Kohler et al., 2011). The KHB method allows us to decompose the total effect of some key variables *X* into direct and indirect effects by examining how they operate through mediator *Z*. The decomposition takes place by comparing the estimated coefficients of *X* in a simplified (reduced form) model, which only includes the key variables and controls, to the estimated coefficient of *X* in the full model, which also includes the mediator variables. The difference between the estimated coefficients in these models amounts to the effect of *X* that is mediated by *Z.* In our analysis, we compared the reduced-form model with the divorce circumstances only (Model 1, same as Table 3, Model 3) to the full model including the divorce circumstances as well as fathers’ involvement and contact frequency (Model 2, 3, and 4).

All models were estimated in STATA 16. In additional analyses (not shown), we compared the estimates with and without control variables but this did not alter the results. For clarity, we present the models with control variables only. In interpreting the results, it must be kept in mind that when we refer to “effects” in the analyses, the term is meant in a statistical sense, as our models only demonstrate approximations of causal effects. Changing the terms ‘effects’ to ‘associations’ would make the discussion of the mediation analysis unclear. Note, however, that for several variables in the analysis, the time order is clear.

For example, conflict refers to the period of marriage whereas father–child contact refers to the situation after divorce.

### Missing Data

There were some missing data in the data set, primarily because of the “don’t know” or “not applicable” option for some of the questions. We were cautious in our coding and recoded “don’t know” and “not applicable” as missing, because there is some uncertainty about what is meant by this. For example, a “don’t know” response to the level of interparental conflict could indicate that the parents did not see each other and had no opportunity to have fights, or the respondent has no recollection. Fathers’ level of education had relatively many “don’t know” answers as well. As we rely on the anchors' reports for information about their fathers, there might be some selection as to which anchors have a missing value on their father’s level of education. To know the father’s level of education, anchors need to know about their father’s past, which could be more difficult for those who do not know their fathers so well, resulting in missing values. Indeed, respondents who had a missing value for their father’s level of education were more likely to have less involved fathers, lower contact frequencies and they were less close to their fathers in adulthood.

To deal with missing data, we used the multiple imputations by chained equations (MICE) approach to impute these values. The MICE algorithm estimates a model for each variable with missing values, with each cycle including the previous estimates to the next model in an iterative process (Royston, 2004, 2005). It predicts missing values based on data of the same respondent and the pattern of missing data (Donders et al., 2006). We performed 25 imputations, after which the estimates of each model were pooled into a single set of estimates and standard errors using Rubin’s rule (Rubin, 1987). All models and tables are based on the same sample size.

## Results

### Descriptive Findings

**Fig. 2:**
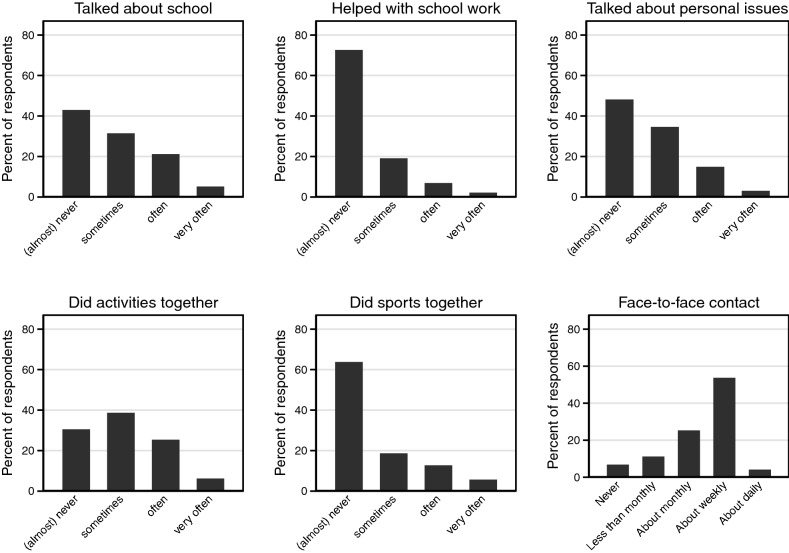
Nonresidential father's involvement and contact frequency with children in youth. *Source*: OKiN Anchor 1.1 UvA 2017, own calculations

We begin by describing the father–child relationship in terms of fathers’ involvement, contact frequency, and closeness in adulthood (Table 1). Figure 2 visualizes the different indicators of the mediator variables of nonresidential fathers’ involvement and face-to-face contact frequencies. It demonstrates that nonresident fathers were particularly unlikely to help with schoolwork or to do sports together. The mean score of fathers’ involvement was 1.73, which indicates a rather low level of fathers’ involvement. Children reported high contact frequencies in youth after divorce, as half of the children (53.5%) saw their fathers about weekly and some (3.9%) even daily, although 6.6% never saw their fathers in youth. It must be noted, however, that the categories are not extremely precise, such that both children who saw their father three times a week may report seeing their father weekly, as well as children who saw their father only once a week, or even biweekly. Nonetheless, it indicates that contact frequency is quite high after divorce. With regards to how close fathers and children were reported to be in adulthood, about four in ten (37.1%) adult children describe their relationship with their father as close or very close, and one in four (25.0%) as reasonably close. This indicates that most adult children have a good relationship with their fathers.

As can be found in Table 1, the mean score of interparental conflict is 2.3 on a scale of (1) to (4), indicating relatively high levels of conflict. The majority of the fathers (72.9%) have been in a cohabiting relationship after their divorce. The average geographical distance between fathers and children is 21.9 km, but the majority (55.1%) live within a 10 km distance of each other. This is lower than more recent findings from the Netherlands that show that 60.8% of parents who divorced live within 5 km of each other in the years following divorce (van der Wiel et al., 2021), which could be explained by the increase in co-parenting in the past decades (Poortman & van Gaalen, 2017).

There were somewhat more females than males in the sample, namely 55.0%. Most anchors had siblings (80.7%). A small majority experienced parental divorce between the age of seven and seventeen (55.9%). At the moment of the questionnaire, the average age of the respondents was 32.1 years old. Most anchors had a partner themselves (88.9%), and about half had children (51.2%). In terms of the level of education, most anchors had completed higher secondary education (44.7%), and the second largest group completed a bachelor's, master’s, or doctoral degree (35.8%). Most fathers (46.3%) fall in the group that has completed primary and lower secondary education, and 29.7% have completed a bachelor, master, or doctoral or equivalent education. The majority of mothers were in a new partnership during the anchor’s youth.

### Direct Effects of Divorce Circumstances on Fathers’ Involvement

In Table 2, we examined whether the divorce circumstances affect fathers’ involvement and contact frequency (path a). These models assess whether there is an association between the key independent variables and the mediators by treating fathers’ involvement and contact frequency as dependent variables. Table 2 reports the results from the ordinary least square regression models for father’s involvement in Model 1, and contact frequencies in Model 2.

The level of interparental conflict produced a similar and significant effect on both fathers’ involvement and contact frequency. We expected a negative effect of interparental conflict on fathers’ involvement and contact frequencies. Children whose fathers had higher levels of conflict with their ex-partner reported significantly lower father involvement by −0.16, or 26% of a standard deviation of father’s involvement (i.e., 0.16 / 0.6). The level of contact frequency decreased significantly with −0.28, which constitutes 21% of a standard deviation of contact frequency (i.e., 0.28 / 1.3). These findings confirm hypotheses 2a and 2b, which holds that the more conflict parents had in the period following divorce, the less involved fathers were in their children’s lives after divorce, and the less frequently fathers and children saw each other.

We found some significant effects for new partnerships on the fathers’ involvement and contact frequencies. We expected that having a new partner would lead to less father involvement and lower contact frequencies. Fathers who had a new partner appeared to be somewhat less involved than fathers who remained single, as involvement reduced by 0.10 (16% of a st. dev. of involvement). An association in the same direction was found between a father’s new partnership and the frequency of contact, as the presence of a new partner reduced contact frequencies significantly with 0.15 (11% of a st. dev. of involvement). These findings suggest that a new partner could be associated with less father–child interaction, thus confirming hypotheses 3a and 3b. In comparing the effects of interparental conflict and new partnerships on father involvement and contact frequency, involvement appears to be somewhat more strongly affected than the frequency of contact.

We expected that fathers who lived further away from their adolescent children would be less involved and would have lower contact frequencies. We found a significant effect of distance on contact frequencies. Fathers who lived more than 10 km away from their children saw their children less frequently with 0.33, which is 25% of a standard deviation of contact frequency. As such, in line with hypothesis 4b, fathers who lived further away are found to have less frequent face-to-face contact with their children. Contrary to our expectations, we found only small and insignificant effects of geographical distance on father involvement (hypothesis 4a). This indicates that differences in geographical distance between parents do not alter fathers’ ability to be involved in their children’s lives, and fathers who live far away are not more or less involved.

The control variables of having siblings, the age at separation, and the father’s level of education had somewhat different outcomes for involvement and contact. Children who had siblings and who were older when their parents separated reported somewhat lower involvement from their fathers, but no significant association was found for how often father and child saw each other. The father’s level of education had a significant positive effect on fathers’ involvement, but not on contact frequency. This means that there is not a great difference between fathers of different levels of education in how often they see their children after divorce, but there is in terms of how involved they are. We found no significant associations of gender and a mother’s new partnerships on both forms of father–child interactions.

### Explaining Father–Child Closeness in Adulthood

Table 3 shows the direct effects of fathers’ involvement, contact frequency, and divorce circumstances on the degree of closeness between fathers and adult children. To assess whether the mediators affected the outcome variable of closeness (path b), we included the effect of fathers’ involvement and father–child contact frequencies as independent variables on closeness in adulthood in Models 1 and 2, respectively. We expected that more father involvement and more frequent contact in youth would have a positive effect on closeness in adulthood. With regards to fathers’ involvement in youth, we found a strong and significant positive effect, which means that the more involved a father was in youth, the closer fathers and children are later on. We also found a significant positive of contact frequency in youth on closeness later on. Children who saw their fathers more often in childhood were closer to their fathers in adulthood. Therefore, hypothesis 1a and b are confirmed.

**Table 3:** Estimates from OLS models of nonresidential fathers’ involvement and contact frequency in youth, and divorce circumstances on the closeness between adult children and fathers. *Source*: OKiN Anchor 1.1 UvA 2017

	Model 1	Model 2	Model 3
Post-divorce father’s involvement	1.045**		
	(25.47)		
Post-divorce contact frequency		0.298**	
		(13.48)	
Interparental conflict before divorce			−0.278**
			(−8.61)
Father's new partner in youth (ref = none)			
Had a new partner			−0.234**
			(−3.47)
Distance between parents (ref. = less than 10 km)			
Over 10 km			−0.068
			(−1.10)
Gender (ref. = female)			
Male	0.060	0.104^~^	0.086
	(1.16)	(1.81)	(1.46)
Anchor’s number of siblings	0.018	−0.022	−0.010
	(0.74)	(−0.85)	(−0.36)
Age at separation (ref. = age 0–6)			
Age 7 or older	0.115*	0.054	0.012
	(2.18)	(0.92)	(0.19)
Current age anchor (centered)	−0.009	−0.018**	−0.021**
	(−1.59)	(−2.93)	(−3.50)
Anchor's partner (ref. = no partner)			
Has a partner	−0.141	−0.142	−0.122
	(−1.53)	(−1.39)	(−1.17)
Anchor's children (ref. = none)			
Has children	0.184**	0.144*	0.161*
	(3.14)	(2.23)	(2.42)
Anchor's level of education (ref. = ISCED 0–2)			
ISCED 3–5	−0.053	−0.048	0.008
	(−0.76)	(−0.63)	(0.10)
ISCED 6–8	−0.108	−0.075	0.034
	(−1.45)	(−0.91)	(0.41)
Father’s level of education (ref. = ISCED 0–2)			
ISCED 3–5	0.012	0.168*	0.113
	(0.16)	(2.06)	(1.33)
ISCED 6–8	0.069	0.327**	0.233**
	(0.95)	(4.12)	(2.72)
Mother’s new partner in youth (ref. = none)			
Had a new partner	0.019	0.015	0.014
	(0.32)	(0.22)	(0.20)
Constant	1.038**	1.865**	3.633**
	(7.76)	(12.96)	(21.93)
N	1948	1948	1948

*t* statistics in parentheses

^~^*p* < 0.10, *p < 0.05, **p < 0.01

In Model 3, we examined the total impact of the divorce circumstances on how close fathers and children were in adulthood (path c), i.e., the effects without controlling for post-divorce involvement and contact. We found significant effects of interparental conflict and new partnerships. We expected that higher levels of interparental conflict would reduce the closeness in the father–child relationship in adulthood. We found that children whose divorced parents had higher levels of conflict were found to have .28 less closeness in adulthood, which is 22% of a standard deviation of closeness (i.e., 0.28 / 1.29). Thus, high levels of interparental conflict appear to have a negative long-term effect on the father–child relationship, which confirms hypothesis 2c.

New partnerships were expected to weaken the father–adult child relationship. Confirming hypothesis 3c, we found a significant negative effect of the presence of a new partner, with children whose fathers had a new partner in youth having .23 less closeness in adulthood. In other words, when a father had a new partner, the relationship between the father and child appears to be less close later on.

Contrary to our expectations, we did not find a significant long-term effect of geographical distance. We expected that fathers who lived more than 10 km away from their children in youth would be less close to their children later on. However, the results show a small and insignificant effect, which means that proximity to a father in youth does not appear to have a lasting influence on the father–child relationship. As such, hypothesis 4c cannot be confirmed.

### Mediation Analyses

In Table 4, we performed a mediation analysis to examine whether the effects of the divorce circumstances on father–child closeness in adulthood are explained by their effect on fathers’ opportunities to be involved. In these models, fathers’ involvement and contact frequency are treated as mediators. The level of interparental conflict was expected to lower father involvement during childhood, and, through this, the possibility to build a close relationship later on. For both father involvement and contact frequency, we found partial mediation. Specifically, 55% of the long-term effect of the level of interparental conflict disappeared when fathers’ involvement was included, and 26% of the effect disappeared when contact frequency was added. When both father involvement and contact frequency in youth were included in the model, the effect of interparental conflict decreased by 62%. As such, there is clear evidence that the negative effect of conflict between parents in youth on father–child closeness in adulthood is partly the result of the way conflict affects father involvement and contact frequencies during childhood. This confirms hypothesis 2d.

**Table 4:** Mediation analysis (KHB) of nonresidential fathers’ involvement and contact frequency in youth on the closeness between adult children and fathers. *Source*: OKiN Anchor 1.1 UvA 2017

	Model 1	Model 2	Model 3	Model 4
Interparental conflict before divorce	−0.278**	−0.124**	−0.207**	−0.107**
	(−8.61)	(−4.19)	(−6.46)	(−3.62)
Father’s new partner in youth (ref = none)				
Had a new partner	−0.234**	−0.144*	−0.196**	−0.136*
	(−3.47)	(−2.43)	(−3.02)	(−2.31)
Distance between parents (ref. = less than 10 km)				
Over 10 km	−0.068	−0.032	0.017	−0.000
	(−1.10)	(−0.59)	(0.29)	(−01)
Post-divorce father’s involvement in youth		0.990**		0.914**
		(23.28)		(20.25)
Post-divorce contact frequency in youth			0.262**	0.104**
			(11.55)	(4.79)
Age at separation (ref. = age 0–6)				
Age 7 or older	0.012	0.063	−0.014	0.049
	0.19)	(1.17)	(−0.24)	(0.92)
Age anchor (centered)	−0.021**	−0.007	−0.016**	−0.006
	(−3.50)	(−1.32)	(−2.64)	(−1.11)
Gender (ref. = female)				
Male	0.086	0.036	0.062	0.030
	(1.46)	(0.69)	(1.09)	(0.58)
Anchor's partner (ref. = no partner)				
Has a partner	−0.122	−0.131	−0.124	−0.131
	(−1.17)	(−1.44)	(−1.23)	(−1.45)
Anchor's children (ref. = none)				
Has children	0.161*	0.167**	0.121^~^	0.151**
	(2.42)	(2.88)	(1.89)	(2.60)
Anchor’s number of siblings	−0.010	0.022	−0.012	0.019
	(−0.36)	(0.93)	(−0.46)	(0.80)
Anchor’s level of education (ref. = ISCED 0–2)				
ISCED 3–5	0.008	−0.047	−0.043	−0.063
	(0.10)	(−0.69)	(−0.57)	(−0.93)
ISCED 6–8	0.034	−0.090	−0.055	−0.116
	(0.41)	(−1.22)	(−0.67)	(−1.57)
Father's level of education (ref. = ISCED 0–2)				
ISCED 3–5	0.113	0.018	0.153^~^	0.042
	(1.33)	(0.24)	(1.88)	(0.55)
ISCED 6–8	0.233**	0.061	0.275**	0.091
	(2.72)	(0.83)	(3.43)	(1.25)
Mother’s new partner in youth (ref. = none)				
Had a new partner	0.014	0.032	0.022	0.033
	(0.20)	(0.53)	(0.33)	(0.56)
Constant	3.633**	1.564**	2.648**	1.329**
	(21.93)	(9.08)	(14.37)	(7.43)
Mediated effects				
Level of interparental conflict		−0.148**	−0.064**	−0.162**
Father’s new partnership		−0.106*	−0.036	−0.113*
N	1948	1948	1948	1948

*t* statistics in parentheses

^~^*p* < 0.10, *p < 0.05, **p < 0.01

The effect of the presence of a new partner on closeness in adulthood was expected to be mediated by father involvement and contact frequency as well. The presence of a new partner had a negative effect on father involvement and closeness, and this effect was significantly reduced by 38% when father involvement was included in the model. In the mediation analysis of contact frequency, the negative effect of a new partner on adult closeness was reduced by 16%, although this was not significant. When both mediator variables were included, the effect of new partnerships decreased significantly by 42%. These findings suggest that the effect of having a new partner is partly explained by how the presence of this new partner affects a father’s involvement immediately after divorce. As such, hypothesis 3d is confirmed.

As there was no direct effect of geographical distance, we did not include geographical distance in the mediation analysis (hypothesis 4d is not applicable).

### Control Variables

We discuss the effect of the control variables on nonresident father–child closeness in adulthood based on the full model, which includes both mediator variables (Table 4, Model 3). We expected that some of the control variables would be relevant for predicting the father–adult child relationship, but interestingly, the only variable that significantly affected how close fathers and children were in adulthood was whether anchors had children themselves. Anchors who had children themselves reported having closer relationships with their fathers than anchors who did not have children. This suggests that the presence of grandchildren brings people closer together. The insignificant effects of the other control variables on father–adult child closeness could be explained by their structural nature, such as the number of full siblings, which is (generally) stable before and after the divorce.

## Conclusions and Discussion

As a result of the increased diversity of family structures in most western societies, fewer children grow up with both parents in the same household (Thomson, 2017). This study draws attention to the role of nonresidential fathers in children’s lives, both in the period following divorce, as well as in adulthood. Building on prior studies, we examined whether there are long-term effects of the circumstances and decisions made following divorce on the relationship between fathers and children in adulthood. We extended prior studies by testing how these long-term effects can be explained by nonresidential father involvement and contact frequencies in youth after the divorce.

The results of this study demonstrate that the level of interparental conflict and the presence of a new partner in a father’s life after divorce negatively affect the degree of closeness between father and child in adulthood. We found that adult children who experienced higher levels of interparental conflict in youth were less close to their fathers in adulthood. This is in line with previous studies and suggests that early interparental conflict functions as a barrier to developing strong relationships in adulthood (Kalmijn, 2015a; Uphold-Carrier & Utz, 2012). Similarly, fathers who had a new partner in youth were less close to their children in adulthood. This indicates that fathers’ commitment to a new partner sometimes comes at the cost of the level of commitment to children, which is in line with previous studies that found that fathers’ repartnering facilitates ties to stepchildren, but not ties to their biological children (Furstenberg et al., 1983; Kalmijn et al., 2019; Manning & Smock, 2000; Manning et al., 2004).

Our mediation analysis demonstrated that the effects of interparental conflict and new partnerships on adult closeness were mediated by father involvement and contact frequency during childhood. In other words, it is partly through the ‘immediate’ effects that interparental conflict and new partners have on fathers’ involvement that fathers and children become less close later in life. This means that in some circumstances fathers’ active involvement and frequent face-to-face contact may help to overcome the negative effects of divorce, although not entirely, as some effects remain.

The persistent effects of interparental conflict and new partners point to their disruptiveness, regardless of how divorce was dealt with. This is partly explained by their nature: also adult children whose parents were continuously married reported lower levels of closeness in case of high levels of conflict (Booth & Amato, 1994). Furthermore, there is evidence of spillover effects of parental marital conflict into conflict in the broader family system (Gerard et al., 2006). The presence of a new partner has been characterized as a form of cumulative instability, as a child that has adjusted to a parental divorce has to adjust to a stepparent figure as well (Raley & Sweeney, 2020). A possible explanation might be that, as a result of divorce, children develop insecure attachment styles, which lead to lower levels of attachment to parents later in life (Woodward et al., 2000).

Contrary to our expectations, the geographical distance between nonresidential fathers and children was not associated with the degree of closeness between fathers and children in adulthood. As a robustness check, we performed three additional analyses (not shown) to test whether different codings of geographical distance would alter our findings. We examined distance as a continuous variable, its natural log, and as a categorical variable with four groups. None of these variables resulted in a different outcome, however, as the non-significant effect remained. These findings suggest that the geographical distance at which a father lives does not reflect the efforts someone is willing to make. A possible explanation is the Dutch context, as the Netherlands is a small country and distances are generally short and relatively easy to overcome. As such, we can conclude that geographical distance as part of the opportunity structure of interaction has no obvious long-term implications on the father–child relationship. Note that there was an effect of distance on contact after divorce, as well as an effect of contact on closeness later on, but the two paths were apparently not strong enough to produce an overall effect of distance in closeness.

Although the examination of father–child contact frequency and father involvement produced largely the same results with regards to the closeness between fathers and children in adulthood, there were some important differences in youth. Most notable were the different outcomes for geographical distance, which was associated with contact frequency but not with involvement. These findings suggest that while it is possible to remain involved over a longer distance, it is a greater challenge to maintain face-to-face contact. To assess whether this is the case, we examined father involvement in more detail. We estimated multivariate ordered logistics regression for each indicator of involvement (not shown), which showed that the likelihood of doing sports together (−0.29, *p* = .013) was significantly lower when fathers lived more than ten kilometers away, whereas school related or personal issues, and doing activities together were not significantly affected. Possibly, telephone contact, and nowadays even more technological advances, such as the smartphone, have enhanced fathers’ possibilities to be involved over a distance, whereas indicators of involvement that concern more regular activities, such as sports, overlap with face-to-face contact and are more feasible from a short distance. These findings corroborate earlier studies demonstrating that time spent and actual involvement (like helping out) are two distinct aspects of parent–child solidarity (Bengtson & Roberts, 1991), which affect the father–child relationship differently (Adamsons, 2018; Adamsons & Johnson, 2013; Amato & Gilbreth, 1999).

In assessing the findings of this study, some limitations should be considered. While the use of retrospective data enabled us to examine the long-term effects of the conditions in the post-divorce period, a disadvantage is that there might be some bias in the responses, as retrospective reports have some measurement errors (de Vries & de Graaf, 2008; de Vries et al., 2009). There are two types of errors: random errors and systematic errors. The effect of the random error is not completely clear, however, because this bias may lead to both underestimations and overestimations. The systematic error often leads to overestimation, because the way someone looks back on their childhood might be influenced by events that took place later in the life course. We cannot rule out that a high-quality relationship in the present could contribute to a more positive memory of the past (Karney & Coombs, 2000). However, in general, this form of retrospective bias is not so great that it invalidates all retrospective data, particularly for concepts that have been shown to be sufficiently valid, such as conflict (Hardt & Rutter, 2004). Nonetheless, the results must be interpreted with some caution. Future studies could overcome this possible bias by collecting data over a longer period of time. Another limitation concerns the specific Dutch context. In the Netherlands, distances are relatively short, because of the small and densely populated geographical area. With a relatively good public transport system and the common use of bicycles, children are able to bridge a distance of 10 km by themselves, whereas in other countries this might be less feasible. It would be interesting to compare different national contexts in future research.

By combining detailed retrospective survey data with geographical data, we made important contributions to understanding the impact of circumstances that characterize the post-divorce period on the long-term father–child relationship. Although there have been important contextual changes compared to the period covered in this study, such as the increasing number of parents that opt for a co-parenting arrangement (Poortman & van Gaalen, 2017), this study provides important insights into the immediate and long-term effects of divorce circumstances on the father–child relationship. The outcomes of proximity for father–child contact frequency, for example, could be taken into account by social services or family courts when deciding on residential related matters. All in all, our study highlights the importance of considering divorce circumstances when examining nonresidential father–child relationships, which could ultimately help avoid children to suffer the consequences of their parents’ divorce.

## Data Availability

The data that support the findings of this study are available as a DANS-KNAW public-use data file.

## Code Availability

The STATA code is available at the Netherlands Interdisciplinary Demographic Institute.
